# Diagnostic criteria for temporomandibular disorders (DC/TMD) for children and adolescents: An international Delphi study—Part 1‐Development of Axis I

**DOI:** 10.1111/joor.13175

**Published:** 2021-05-19

**Authors:** Roberto Rongo, EwaCarin Ekberg, Ing‐Marie Nilsson, Amal Al‐Khotani, Per Alstergren, Paulo Cesar Rodrigues Conti, Justin Durham, Jean‐Paul Goulet, Christian Hirsch, Stanimira I. Kalaykova, Flavia P. Kapos, Osamu Komiyama, Michail Koutris, Thomas List, Frank Lobbezoo, Richard Ohrbach, Christopher C. Peck, Claudia Restrepo, Maria Joao Rodrigues, Sonia Sharma, Peter Svensson, Corine M. Visscher, Kerstin Wahlund, Ambra Michelotti

**Affiliations:** ^1^ Department of Neurosciences, Reproductive Sciences and Oral Sciences School of Orthodontics University of Naples Federico II Naples Italy; ^2^ Department of Orofacial Pain and Jaw Function Faculty of Odontology Malmö University Malmö Sweden; ^3^ Center for Oral Rehabilitation FTV Östergötland Norrköping Sweden; ^4^ Scandinavian Center for Orofacial Neurosciences Sweden; ^5^ East Jeddah Hospital, Ministry of Health Jeddah Saudi Arabia; ^6^ Department of Dental Medicine Karolinska Institute Huddinge Sweden; ^7^ Department of Prosthodontics and Periodontology Bauru School of Dentistry – University of São Paulo Bauru Brazil; ^8^ Bauru Orofacial Pain Group University of São Paulo Bauru Brazil; ^9^ School of Dental Sciences Newcastle University Newcastle Upon Tyne UK; ^10^ Newcastle Upon Tyne Hospitals NHS Foundation Trust Newcastle Upon Tyne UK; ^11^ Faculty of Dental Medicine Laval University Quebec QC Canada; ^12^ Clinic of Pediatric Dentistry University of Leipzig Leipzig Germany; ^13^ Department of Oral Function and Prosthetic Dentistry College of Dental Sciences Radboud University Medical Center Nijmegen The Netherlands; ^14^ Department of Epidemiology University of Washington Seattle WA USA; ^15^ Division of Oral Function and Rehabilitation Nihon University School of Dentistry at Matsudo Matsudo Japan; ^16^ Department of Orofacial pain and Dysfunction Academic Centre for Dentistry Amsterdam (ACTA University of Amsterdam and Vrije Universiteit Amsterdam Amsterdam The Netherlands; ^17^ Department of Oral Diagnostic Sciences University at Buffalo Buffalo NY USA; ^18^ Faculty of Medicine and Health The University of Sydney Sydney NSW Australia; ^19^ CES‐LPH Research Group Universidad CES Medellin Colombia; ^20^ Institute for Occlusion and Orofacial Pain Faculty of Medicine University of Coimbra Coimbra Portugal; ^21^ Section of Orofacial Pain and Jaw Function School of Dentistry and Oral Health Aarhus Denmark; ^22^ Department of Stomatognathic Physiology Kalmar County Hospital Kalmar Sweden

**Keywords:** adolescents, children, Delphi study, diagnostic criteria, temporomandibular disorders

## Abstract

**Background:**

Since in children and adolescence prevalence is assessed mainly on self‐reported or proxy‐reported signs and symptoms; there is a need to develop a more comprehensive standardised process for the collection of clinical information and the diagnosis of TMD in these populations.

**Objective:**

To develop new instruments and to adapt the diagnostic criteria for temporomandibular disorders (DC/TMD) for the evaluation of TMD in children and adolescents.

**Method:**

A modified Delphi method was used to seek international consensus among TMD experts. Fourteen clinicians and researchers in the field of oro‐facial pain and TMD worldwide were invited to participate in a workshop initiated by the International Network for Orofacial Pain and Related Disorders Methodology (INfORM scientific network) at the General Session of the International Association for Dental Research (IADR, London 2018), as the first step in the Delphi process. Participants discussed the protocols required to make physical diagnoses included in the Axis I of the DC/TMD. Thereafter, nine experts in the field were added, and the first Delphi round was created. This survey included 60 statements for Axis I, and the experts were asked to respond to each statement on a five‐item Likert scale ranging from ‘Strongly disagree’ to ‘Strongly agree’. Consensus level was set at 80% agreement for the first round, and at 70% for the next.

**Results:**

After three rounds of the Delphi process, a consensus among TMD experts was achieved and two adapted DC/TMD protocols for Axis I physical diagnoses for children and adolescents were developed.

**Conclusion:**

Through international consensus among TMD experts, this study adapted the Axis I of the DC/TMD for use in evaluating TMD in children and adolescents.

## Graphical abstract

The Delphi process included three round and was used to was used to seek international consensus for Axis I assessment among 23 TMD experts. Thanks to this Delphi study, experts developed new instruments that aim to assess physical diagnoses (Axis I) of TMDs in children and in adolescents, by modifying the DC/TMD for adults.

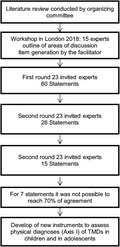

## 1 INTRODUCTION

Temporomandibular disorders (TMDs) are a group of musculoskeletal disorders that involve the temporomandibular joints (TMJs), masticatory muscles and associated tissues or structures.^1^ TMDs are a significant public health problem with a prevalence in adults of 5%–30% according to different pathologies, different age range and different assessment forms.^2^, ^3^, ^4^ Reported TMD prevalence in children and adolescents varies widely in the literature from 4.2% to 68%, depending on population and method of assessment.^5^, ^6^, ^7^, ^8^, ^9^ Painful and dysfunctional TMD might be associated with emotional stress, depression, sleep and hormonal disturbances, and functional complications.^10^, ^11^, ^12^ In turn, the patient's daily life will be adversely affected by the presence of a TMD; in addition, TMDs result in increased pursuit of medical care which consumes both time and money.^13^ TMDs are a considerable health problem in children and adolescents; several studies consistently show that TMD prevalence increases with age from childhood to adolescence,^7^, ^8^, ^9^, ^14^, ^15^ especially for females.^7^, ^8^ However, in children under the age of 10 prevalence is assessed mainly on self‐reported or proxy‐reported signs and symptoms; hence, there is a need to develop a more comprehensive standardised process for the collection of clinical information and the diagnosis of TMD in children and adolescents, so that reliability and validity can be assessed and improved for this population.^16^

Since 2014, the international standard for the assessment of TMDs is the Diagnostic Criteria for TMD (DC/TMD).^1^ The DC/TMD consists of two axes and their respective instruments: Axis I for physical diagnoses and Axis II for assessment of psychosocial status and pain‐related disability. The DC/TMD is validated for several diagnoses as based on a standardised assessment protocol including history and clinical examination. A diagnostic algorithm utilising both history and clinical data permits to have very high sensitivity and specificity for some TMD subgroups and consequently excellent diagnostic accuracy for TMD in adults.^1^

The DC/TMD is validated for individuals who are 18 or more years of age; consequently, using the DC/TMD in children and adolescents requires a form of adaptation for each age group.^1^ Adaptation of the DC/TMD includes (1) a separate language review for both questionnaires and clinical examination, due to the difference in understanding and speaking skills between adults, adolescents and children,^17^ and (2) modified protocols for clinical assessment. Adaptation and assessment of content and construct validity of the Spanish translation of the DC/TMD Axis I were performed in 7‐ to 11‐year‐old children in Colombia, finding high internal consistency (.72 ≤ Cronbach's alpha ≤ .94).^18^

In the development or adaptation of diagnostic systems for which high‐quality evidence is not yet available, relying on experience of international experts in the field to achieve consensus on a topic is an effective method moving towards testable hypotheses.^19^ One of the methods used for decision‐making among experts is the Delphi method. This method includes a series of questions and statements that are regrouped in different ‘rounds’ and thereby presents several advantages over other consensus techniques: it is anonymous and there is less possibility that some experts may influence the opinions of other experts as they might in a face‐to‐face setting.^20^ Moreover, as it is usually performed online, experts from different geographic regions can easily be included. The Delphi method is well recognised as legitimate and suitable for addressing highly complex problems, such as the development of a new diagnostic instrument, and as being flexible and adaptable to different research contexts and data collection.^21^

The aim of this study was to develop new instruments to diagnose TMDs in children and in adolescents by the adaptation of DC/TMD Axis I through an international Delphi study with a consensus among TMD experts. This paper is focused on the Delphi process related to the DC/TMD Axis I, while the Delphi process related to the DC/TMD Axis II and the full examination protocols of the DC/TMD for children and adolescents will be described in future publications.

## 2 MATERIALS AND METHODS

The modified Delphi method was used to seek international consensus for Axis I assessment among TMD experts.^22^ Development of the adaptations of the DC/TMD started at a workshop promoted by the International Network for Orofacial Pain and Related Disorders Methodology (INfORM) at the General Session of the International Association of Dental Research in London in 2018. Fourteen TMD experts (RO, SS, FK, CR, MJR, JD, MK, SK, AM, TL, PA, ECE, IMN, CP) and the Delphi facilitator (RR) participated in the meeting and created a list of key issues, related to the applicability of DC/TMD for children and adolescents. After this workshop, the facilitator (RR), who did not participate in the online Delphi survey, constructed a survey of 60 statements based on the key issues pertaining to physical diagnoses (Axis I) as outlined by the experts. Each of these statements was subsequently assessed during the Delphi online survey. The statements addressed demographics, screening, health, Symptom Questionnaire, clinical examination, imaging, and diagnosis as presented in the DC/TMD.

Twenty‐three experts worldwide (Table 1) were invited by e‐mail to participate in the Delphi process; this included 14 experts who had previously participated in the workshop in London (excluding the facilitator RR) and a further 9 experts who were identified among different competences, such as surgeons, orthodontists, oro‐facial pain specialists, paediatric dentists, physiotherapists, psychologists and epidemiologists; 100% of the invited experts agreed to participate. An expert was defined as a person with at least five years of experience in the clinical management of TMD patients, experience in using the DC/TMD and research interest in TMDs based on publications in international peer‐reviewed journals. The experts were asked to answer each statement on a five‐item Likert scale ranging from ‘Strongly disagree’ to ‘Strongly agree’. In addition, comments could be provided for each statement. Agreement on each statement was reached if the sum of experts replying ‘Agree or Strongly agree’ or the sum of experts replying ‘Disagree or Strongly disagree’ was equal to or higher than the selected threshold for each round. Threshold level for consensus was set at 80% agreement (18 out of 23) for the first round and at 70% (16 out of 23) for the next rounds.^23^, ^24^ The Survey Monkey^®^ cloud‐based software (SVMK) was used to develop the online survey. Together with the invitation to participate in the survey, each expert received a letter of instructions and a list of references with full‐text versions of all the papers.

**TABLE 1:** List of experts included in the Delphi study with area of expertise and affiliations

	Name surname	Area of expertise	Affiliations
1.	Al‐Khotani Amal	TMD/Oro‐facial Pain in children and adolescents; Paediatric Dentistry; Paediatric Psychology, Epidemiology	Ministry of Health (Saudi Arabia)
2.	Alstergren Per^a^	TMD/Oro‐facial Pain; Rheumatological disease; TMJ physiology	Malmö University (Sweden)
3.	Durham Justin^a^	TMD/Oro‐facial Pain; TMD pathophysiology; TMD treatment	Newcastle University (United Kingdom)
4.	Ekberg EwaCarin^a^	TMD/Oro‐facial Pain; TMD pathophysiology; TMD treatment	Malmö University (Sweden)
5.	Goulet Jean‐Paul	TMD/Oro‐facial Pain; TMD treatment; Oral disease	Laval University (Canada)
6.	Hirsch Christian	Epidemiology; TMD/Oro‐facial Pain in children and adolescents; TMD treatment	University of Leipzig (Germany)
7.	Kalaykova Stanimira I.^a^	TMD/Oro‐facial pain; Dental Sleep Disorders; Oral physiology	Radboud University Medical Centre (The Netherlands)
8.	Kapos Flavia P.^a^	TMD/Oro‐facial Pain; Epidemiology; TMD diagnosis	University of Washington (United States of America)
9.	Komiyama Osamu	TMD/Oro‐facial Pain; TMD pathophysiology; TMD treatment	Nihon University (Japan)
10.	Koutris Michail^a^	TMD/Oro‐facial pain; Dental Sleep Disorders; TMD pathophysiology	ACTA (The Netherlands)
11.	List Thomas^a^	TMD/Oro‐facial Pain; Oral physiology; TMD treatment	Malmö University (Sweden)
12.	Lobbezoo Frank	TMD/Oro‐facial Pain; Oral Movement Disorders; Dental Sleep Disorders	ACTA (The Netherlands)
13.	Michelotti Ambra^a^	TMD/Oro‐facial Pain; TMD treatment; Orthodontics	University of Naples Federico II (Italy)
14.	Nilsson Ing‐Marie^a^	Epidemiology; TMD/Oro‐facial Pain in children and adolescents; TMD treatment	Malmö University (Sweden)
15.	Ohrbach Richard^a^	TMD/Oro‐facial Pain; Psychology; Epidemiology	University of Buffalo (United States of America)
16.	Peck Christopher C.^a^	TMD/Oro‐facial Pain; TMD treatment; Neuroscience	University of Sydney (Australia)
17.	Restrepo Claudia^a^	TMD/Oro‐facial Pain in children and adolescents; Paediatric Dentistry; Dental Sleep Disorders	Universidad CES (Colombia)
18.	Rodrigues Conti Paulo Cesar	TMD/Oro‐facial Pain; TMD diagnosis; TMD treatment	Universidade de São Paulo (Brazil)
19.	Rodrigues Maria Joao^a^	TMD/Oro‐facial Pain; Dental Sleep Disorders; TMD treatment	University of Coimbra (Portugal)
20.	Sharma Sonia^a^	TMD/Oro‐facial Pain; Epidemiology; TMD diagnosis	University of Buffalo (United States of America)
21.	Svensson Peter	TMD/Oro‐facial pain; Neuroscience; Oral physiology	Aarhus University (Denmark)
22.	Visscher Corine M.	TMD/Oro‐facial pain; Physiotherapy; Dental Sleep Disorders	ACTA (The Netherlands)
23.	Wahlund Kerstin	Epidemiology; TMD/Oro‐facial Pain in children and adolescents; TMD treatment	Malmö University (Sweden)

^a^ Experts that participated in the workshop in London 2018.

**FIGURE 1:**
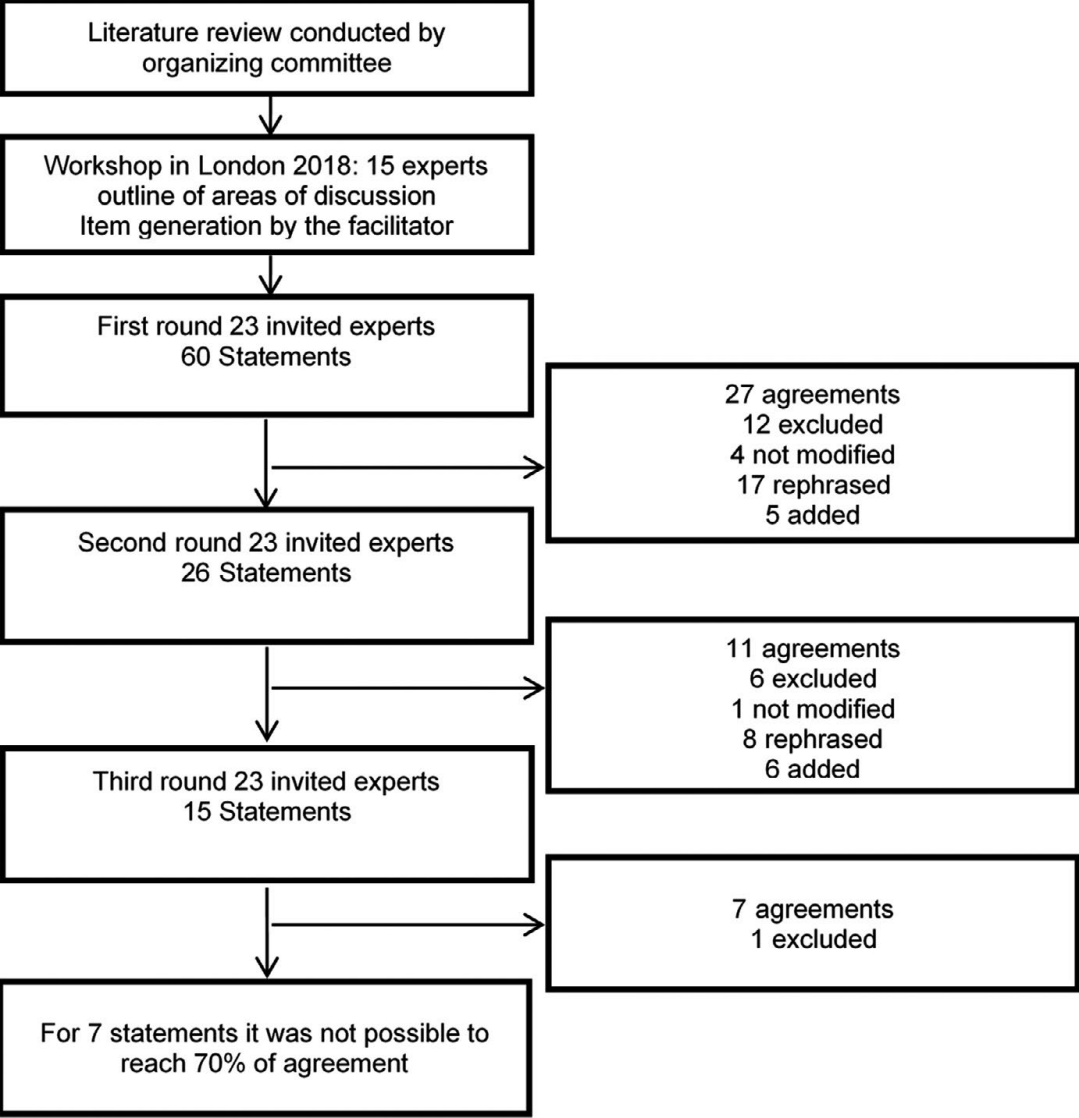
Flow chart of Delphi rounds

The Delphi process is shown in Figure 1; after Delphi round‐1, the facilitator and the organising committee (ECE, IMN, AM) evaluated the results. Based on comments from the experts, existing statements were either rephrased or removed or new statements were added, resulting in a total of 26 statements for Delphi round‐2. A similar process of evaluating the experts’ replies and comments was used at the second round which led to 15 statements for Delphi round‐3. Evaluation of the replies and data analysis was performed blinded; that is, the organising committee (ECE, IMN, AM) did not know the experts’ panels identities. At the end of each round, the experts received a document with the instructions for the next round and a summary of the previous round's evaluation. Only the facilitator (RR) kept the code list to match responses to the experts’ identities. Final consensus was achieved in November 2019. The present manuscript was sent to all the TMD experts who were invited to be co‐authors, and the manuscript was finalised in September 2020.

## 3 RESULTS

The results of the three Delphi rounds are shown in Table 2. The response rate was 100%; that is, all experts responded to all statements in each of the three rounds.

**TABLE 2:** Round of agreement achievement

	Round 1	Round 2	Round 3	No agreement
Structure				
Age	x			
Short and Complete Forms		x		
History				
Screening		x		
Demographics, health questionnaire, Symptom Questionnaire		x		
Clinical examination				
Mandatory commands			x	
Jaw opening			x	
Jaw lateral and protrusive excursions				x^a^
Sounds during jaw opening		x		
Sounds during jaw lateral and protrusive excursions				x^a^
Muscular and joint palpation			x	
Referred pain				x^a^
Familiar pain			x	
Imaging				
Imaging		x		

^a^ Agreement achieved only in adolescents.

Delphi round‐1 resulted in 45% (27 out of the 60 statements) agreement among the experts. Of the remaining 33 statements that did not achieve consensus at Delphi round‐1, 12 statements were excluded, 4 were retained as‐is, 17 were rephrased based on experts’ answers and comments, and 5 new statements were added, for a total of 26 statements presented at round‐2. In the Delphi round‐2, 11 out of 26 statements (42%) reached the 70% agreement. Out of the 15 statements not reaching consensus, 8 were rephrased, 6 were excluded, 1 was retained as‐is, and 6 new statements were added according to the experts’ comments to create a survey of 15 statements presented at round‐3. Finally, in the Delphi round‐3, 7 out of 15 statements (47%) reached the 70% consensus. Out of the 8 statements not reaching consensus, 1 statement with clear disagreement was excluded, and 7 statements, albeit with a trend towards agreement, failed to achieve consensus (Figure 1).

### 3.1 Demographics, screening, health and Symptom Questionnaire

During the Delphi survey, experts agreed to define adolescents from 10 years of age or older. Participants agreed to create two different Axis I protocols: one for children and one for adolescents. For each of the child and adolescent DC/TMD protocols, consensus indicated that both a short version for screening and a comprehensive version needed to be created. There was agreement among the experts to include three general health questionnaires: one for children, one for adolescents and one for their parents, two demographic questionnaires one for children and one for adolescents, and a rephrased form of the Symptom Questionnaire, each adapted for children and adolescents. There was agreement to use the 3Q/TMD questionnaire^25^ as an instrument for TMD screening in both age groups. Finally, the clinical diagnostic classification already present in the DC/TMD was retained.

### 3.2 Clinical examination

The experts agreed not to use the mandatory commands for the clinical examination such as used in the adult version of the instrument but sought to provide instructions for the clinician to explain the concepts included in the DC/TMD.

#### 3.2.1 Adolescents

Experts agreed to maintain the examination of jaw movements (opening, closing, protrusion and laterotrusion) including the report of pain on movement, as it is used in the protocol in the adult version, using cut‐off measurements for limited opening already present in the literature (≤36 mm, 3rd percentile at 10 years of age).^26^ Muscle and joint palpation pain and the evaluation of joint noises were not modified with respect to the adult version. Indeed, regarding the muscle and TMJ pain assessment, the experts agreed to maintain both the 30‐day time frame as the default period for symptoms relevant to the diagnosis and the amount of pressure as recommended by the DC/TMD for adult, and they agreed to ask for familiar and referred pain. Agreement was also achieved among experts to maintain the examination of joint noises during all mandibular movements, as it is in the DC/TMD for adults.

#### 3.2.2 Children

Experts agreed only to maintain the examination of jaw opening and closing movements, including pain on movement, as it is in the DC/TMD for adults, using cut‐off measurements for limited opening (≤32 mm, 3rd percentile at 6 years of age).^26^ No agreement, however, was obtained in retaining or not the assessment of lateral and protrusive movements.

Regarding muscle and joint palpation, the recommendations for children were to assess muscle pain by palpation of the masseter and temporalis at three sites instead of nine with the usual recommended load of 1 kg/cm^2^. Palpation of supplementary masticatory muscles was considered optional. The TMJ palpation was kept as it is in the adult version. However, while there was consensus in asking for familiar pain in children, the threshold of the 70% of agreement was not achieved on whether or not to include the assessment of referred pain, and what should be considered the correct time frame to identify familiar pain in children. Finally, the experts agreed to evaluate joint noises during opening and closing movements, but there was not consensus on considering as supplementary the joint noise assessments during the lateral and protrusive movements.

### 3.3 Imaging

The experts agreed on considering imaging as a supplementary test in selected cases, such as those with no clear diagnosis, needing confirmation of a diagnosis, or with a differential diagnosis.

## 4 DISCUSSION

In light of the prevalence of TMD in children and adolescents, the aim of this Delphi study was to collect and organise expert opinions to develop a standardised protocol for TMD diagnosis in children and adolescents by adapting the pre‐existing DC/TMD for adults. The Delphi method was used to reach a consensus among TMD experts regarding the changes needed in the DC/TMD diagnostic protocol in order to be adopted for use in children and in adolescents.

### 4.1 Demographics, screening, health and Symptom Questionnaire

Adolescents were defined from 10 years of age or older, according to the World Health Organization (WHO) definition.^27^ However, the ability of the individual to understand and respond to the questions is not related only to age. Hence, for individuals transitioning between childhood and adolescence, the child or adolescent assessment protocol should be selected depending on the patients’ cognitive development. Children's cognitive development shows four main stages: sensorimotor period (birth‐2 years of age), preoperational period (2–7 years old), concrete operational period (7 years old‐puberty), formal operations (puberty to adulthood), and although the stages are sequential, their time frame is flexible.^28^, ^29^ The identification of children's cognitive development may be possible through specific tests such as the Differential Ability Scales‐II (DAS‐II)^30^ or the Kaufman Assessment Battery for Children (KABC),^31^ but these tools would increase the time burden of the consultation (around 60 minutes to complete the test). Therefore, in research setting the DC/TMD for children should be used in subjects <10 years old, while the DC/TMD for adolescent in subjects ≥10 years old. In a clinical setting, the clinician may be able to grossly identify the cognitive level of the child during the anamnesis and then select the assessment protocol.

The general health questionnaire, demographic questionnaire and Symptom Questionnaire should be modified for children and adolescents from the DC/TMD protocol. Furthermore, the experts agreed to add a general health questionnaire for the parents. Parents’ health is an important factor for children with acute musculoskeletal pain; more specifically, chronic pain in parents might be a predictor of children's pain intensity and activity limitations related to pain.^32^

As with the DC/TMD for adults, there was agreement in developing both a short and a comprehensive version for each of children and for adolescents, allowing an initial screening evaluation that might be followed by a comprehensive assessment.^1^ The short version would be intended for routine use not only by general dentists but also by other specialists outside the field of dentistry, in order to promote early diagnosis (paediatricians, rheumatologist, etc.)

As screening questionnaires, the TMD pain screener^33^ and the 3Q/TMD questionnaire were considered.^25^ The experts agreed to choose the 3Q/TMD, which includes three questions: two on pain and one on function. The choice was supported by validation of the two pain questions in a Swedish sample of adolescents,^34^ and that the 3Q/TMD included one other question that assesses jaw function. In addition, because the Symptom Questionnaire already includes the TMD pain screener items, there is no real shortcoming in adopting the 3Q/TMD screener for children and adolescents. The INfORM research agenda encourages the assessment of the reliability and validity of the 3Q/TMD in a child and adolescent population by its members in their respective institutions.

### 4.2 Clinical examination

The need to use mandatory commands was much discussed among the experts. Mandatory commands are structured verbal instructions given to the patients prior to and during performing the clinical examination in order to promote maximal reliability. However, the use of mandatory commands represents a high barrier in the implementation of the DC/TMD in daily general practice.^35^ A recent study demonstrated that not using mandatory commands did not affect the diagnostic reliability of pain‐related TMD in Swedish adults in a general dentistry setting.^35^ Therefore, the experts’ final suggestion was to eliminate the mandatory commands and to provide a list of procedural instructions to the examiner, explaining the examination process and the intent of each command in detail. In this way, the examiner could understand the concepts and the intention behind each procedure and then use his/her own words with the child or adolescent patient in a manner that would be tailored to that individual and presumably easy to comprehend.

#### 4.2.1 Adolescents

For the adolescent population, assessment of the range and pain on movements (opening, closing, laterotrusion and protrusion) was retained as it is in the DC/TMD for adults. Albeit time‐consuming, in adults this part of the clinical examination provides additional information, useful in the diagnosis of pain‐related TMD.^1^ The experts agreed to add cut‐off measurements for limited opening according to data reported in the literature with a lower threshold of 36 mm that represents the 3rd percentiles at 10 years of age.^26^

The experts agreed to keep the assessment of joint noises to identify displacement or degenerative TMD as it is in the DC/TMD.

Palpation of the masseter, temporalis and the TMJ were maintained as it is in the DC/TMD version for adults, asking for familiar pain and for referred pain when there is a need to discriminate among the sub‐types of myalgia. For the masticatory muscles, masseter and temporalis, a nine‐point palpation sequence was confirmed, and the same palpation procedure of the DC/TMD for adults was also suggested for the TMJ lateral pole and around the pole. During the palpation examination, the load exerted for muscular palpation and around the pole must be maintained from two up to five seconds with an intensity of 1 kg/cm^2^ in order to evaluate for referred pain, while the same time but with load of 0.5 kg/cm^2^ is retained for lateral pole palpation. During palpation, patients should be asked for familiar pain and referred pain. Familiar pain is defined as ‘pain that is like or similar to the pain that the patient has been experiencing in the last 30 days’.^1^ This concept was found to be fundamental for improving sensitivity and specificity for TMD diagnosis and in particular minimising false positives. Referred pain is defined as ‘pain present outside the boundaries of the assessed tissue’ and is evaluated by maintaining a steady palpation pressure for five seconds, before asking patients about pain spreading/referral. Provoked TMD pain might refer to another anatomical area such as the teeth or neck; hence, this evaluation helps to identify the correct source of pain.^1^

#### 4.2.2 Children

The experts agreed to keep the opening and closing examination for children as it is in the adult version, but there was no agreement for measuring lateral and protrusive movements. Indeed, 65% of the experts (15 out of 23) disagreed on the need to assess mandibular excursions in children due to the difficulty in intentionally performing such movements and the low reliability. The committee decided to retain lateral and protrusive movements in the clinical examination to evaluate the usefulness of such parameters in future research protocols. There was agreement to assess joint noises during the opening and closing movements, while 65% of the experts disagreed with noises assessment during laterotrusion and protrusion. The lack of agreement was due to the poor reliability and the low prevalence of clicking in laterotrusion in children. Nonetheless, the committee recommends assessing joint noises during lateral and protrusive movements while waiting for diagnostic accuracy data in the future. Regarding the muscular palpation in children, the experts agreed to modify the DC/TMD protocol. Due to the small size of masseter and temporalis muscle, and to reduce clinical examination time, the experts agreed to palpate only three points per muscle, one for each area (anterior, middle, posterior bellies for the temporalis, and origin, body, and insertion for the masseter). The time and the load needed to perform the palpation were otherwise retained as in the recommendation for adults. Although agreement was reached on asking for familiar pain, the experts did not agree on the time frame related to familiar pain in children. Time perception in children is different compared to that of adults: until the age of 10 children do not spontaneously use explicit timing‐related strategies, and the first step in the acquisition of time knowledge is completed after 12 years old.^36^, ^37^, ^38^, ^39^ Hence, the use of a time frame related to familiar pain could be complicated or misleading in those ages. Since no agreement was reached, the 30‐day time frame was retained to be tested in future studies. However, it must be considered that a very recent study, published after the end of the Delphi process, tried a 15‐day time frame in the assessment of the Symptom Questionnaire and in the clinical examination of DC/TMD Axis I and found better accuracy compared to the 30‐day time frame in children between 7 and 11 years.^18^ Future studies comparing the 30‐day time frame and the 15‐day time frame should be conducted on this population to further support this hypothesis; in addition, the role of parent information should be considered. Finally, agreement regarding the assessment of referred pain in children was not achieved. Referred pain was considered by the experts as a complex concept to understand and, as far as we know today, does not guide the choice, or change in the management strategy. On the other hand, it might be very important to determine possible severity of pain and possible syndromes associated with such pain. Considering the lack of agreement achieved, the committee decided to test the comprehension of the referred pain by children during future studies.

### 4.3 Imaging

Experts agreed that imaging such as magnetic resonance imaging and/or computed tomography (cone beam or axial) should be performed only when needed. Because children and adolescents are still growing, some TMDs, even if less common, should be considered very important in this population. For example, TMJ manifestations of juvenile idiopathic arthritis,^40^ idiopathic condylar resorption or osseous ankylosis that are present in the expanded taxonomy of the DC/TMD, are related to growth disturbances and may have severe consequences in children.^40^, ^41^, ^42^, ^43^ For most of diagnoses included in the expanded taxonomy, there is still not a high diagnostic performance of the clinical examination; hence, imaging can help in better identifying these pathologies. Therefore, the role of the imaging becomes fundamental in cases of unclear diagnosis or to generate information able to influence the treatment plan, or to follow up the patient.

### 4.4 Delphi study design

The modified Delphi process enabled the creation of expert consensus in setting up new evaluation protocols for the diagnosis of TMD in children and adolescents.^22^ The classical Delphi is a useful forecasting tool based on iterative sequential individual expert input, and in the modified Delphi groups of experts are called to make decisions simultaneously.^22^ The Delphi group consisted of 23 international experts from Europe, North and South America, Asia and Oceania. The wide dissemination of the DC/TMD for adults through the translation into almost 20 languages made this project possible.

The ideal number of experts that should be included in a Delphi process is not established. In planning this kind of study, there is a delicate balance between the amount of information that a large number of participants might produce and the difficulty in analysing data and reaching agreement. In this study, 23 world experts were included, representing professionals involved in TMD diagnosis and treatment, who provided global perspectives on what can be considered useful for TMD diagnosis.^44^ This Delphi process started with a face‐to‐face meeting involving 14 of the 23 who were invited in London to create a list of key issues, related to the applicability of DC/TMD for children/adolescents, to be used by the facilitator to develop the survey. This approach allowed the facilitator to create a survey based on the experts’ suggestions, avoiding underestimating or overestimating the importance of some aspects due to personal opinions.

To complete DC/TMD Axis I for children and adolescents, three rounds were necessary, and the level of agreement was set for the Delphi round‐1 at 80% and for the Delphi round‐2 and round‐3 at 70%. There is not a defined cut‐off for agreement in Delphi studies (51%,^45^ 70%^23^ or 80%^24^); on the other hand, it has been shown that changing the cut‐off from 80% to 70% does not influence the results if new questions are proposed.^46^ The Delphi was stopped after three rounds because it was clear, after the analysis of comments from the participants, that some points concerning children's clinical examination needed to be tested through a content and criterion validation study.

The construction of the DC/TMD for adolescents and for children also needs the development and adaptation of the instruments to evaluate psychosocial status and pain‐related disability (Axis II). For the assessment of Axis II, the DC/TMD includes questionnaires that evaluate jaw function and oral behaviours, and that screen for depression, anxiety and other comorbidities. In order to have a wide information on psychological aspects, the committee decided to also include experts in psychosocial disciplines and to create a new Delphi study. This new Delphi comprising world experts in psychological constructs for children and adolescents and oro‐facial pain experts aims to adapt the Axis II of the DC/TMD for adults, and the results of this Delphi process will be presented in future publications.

## 5 CONCLUSIONS AND FUTURE DIRECTIONS

Thanks to this Delphi study, experts developed new instruments that aim to assess physical diagnoses (Axis I) of TMDs in children and in adolescents, by modifying the DC/TMD for adults. The developed instruments need to be validated.

To complete the creation of DC/TMD for children and DC/TMD for adolescents, a new Delphi study was conducted for the development of instruments to evaluate the psychosocial status and pain‐related disability within DC/TMD Axis II, that will be presented in a separate paper.

Once that both axes were adapted for children and adolescents, other papers will describe the short and comprehensive form of the child DC/TMD and adolescent DC/TMD, including the developed instruments.

## CONFLICT OF INTEREST

The authors declare that they have no conflict of interest.

## AUTHOR CONTRIBUTION

RR, ECE, IMN, AM Conception and design of study; RR, ECE, IMN, AM Acquisition of data; RR, ECE, IMN, AM Data analysis and/or interpretation; AAK, PCRC, JPG, CH, OK, FL, PS, CMV, KW, RO, SS, FK, CR, MJR, JD, MK, SK, AM, TL, PA, ECE, IMN, CP, RR Drafting of manuscript and/or critical revision AAK, PCRC, JPG, CH, OK, FL, PS, CMV, KW, RO, SS, FK, CR, MJR, JD, MK, SK, AM, TL, PA, ECE, IMN, CP, RR Approval of final version of manuscript.

## Data Availability

The data that support the findings of this study are available from the corresponding author [RR], upon reasonable request.
